# Improvement in Heavy Metal Removal from Wastewater Using an External Magnetic Inductor

**DOI:** 10.3390/nano9111508

**Published:** 2019-10-23

**Authors:** Fernanda Lyzeth Rivera, Francisco Javier Palomares, Pilar Herrasti, Eva Mazario

**Affiliations:** 1 Departamento de Química Física Aplicada, Facultad de Ciencias, Universidad Autónoma de Madrid, Francisco Tomás y Valiente 7, 28049 Madrid, Spain; fernanda.rivera@estudiante.uam.es; 2 Instituto de Ciencia de Materiales de Madrid, ICMM/CSIC, Sor Juana Inés de la Cruz 3, 28049 Madrid, Spain; fjp@icmm.csic.es

**Keywords:** magnetic inductor, hot spots, magnetic harvesting, water treatment, heavy metals, pollutant, adsorption

## Abstract

Magnetite nanoparticles (Fe_3_O_4_) of 12 ± 4 nm diameter are electrochemically synthesized for the adsorption and magnetic harvesting of Cr(VI) from contaminated simulated solutions. The removal of Cr(VI) from aqueous media follows pseudo-second-order kinetics. The adsorption efficiency is evaluated in three different scenarios. In standard conditions, i.e., at room temperature; in a thermal bath working at 60 °C, where the temperature could be considered homogeneous within the solution; and finally, under magnetic induction heating, while adjusting the frequency and magnetic field used to attain the same temperature as in the bath experiments. Two benefits of using a magnetic inductor are demonstrated. First, the removal efficiency is almost doubled in comparison to that of the room temperature experiments, and it is higher by 30% compared to that of the bath setup. At the same time as the adsorption occurs, a redox reaction occurs on the surface of the nanoparticles, and Cr(VI), the predominant species in the contaminated solution, is significantly reduced to Cr(III). Through X-ray photoelectron spectroscopy, it is shown that a greater reduction effect is achieved when working in induction conditions than at room temperature. This is the first time that this synergistic effect using magnetic induction heating has been demonstrated for heavy metal decontamination of wastewater.

## 1. Introduction

Currently, pollution has become a worldwide problem of great concern due its harmful effects on the environment and human health [1]. Heavy metals such as lead, cadmium, mercury, arsenic, silver, chromium, and copper are dangerous pollutants because they cannot be degraded. These metals are released to the environment by natural and anthropogenic processes, such as in mining [2], cosmetics [3], and the aerospace industry [4]. Among the different types of pollution, water contamination is considered one of the most severe, and efforts to reverse the consequences on human health cost millions of dollars every year [5]. There is a risk that 1/3 of the world’s population will suffer from a lack of potable water in 2030 [6]. Among the heavy metals, hexavalent chromium (Cr(VI)) is considered one of the most toxic because it has mutagenic and teratogenic properties [7]. It can cause adverse effects on mammal organs and it is a carcinogenic agent even at low concentrations [8]. Cr(VI) is soluble in water and can be found as CrO_4_^2−^, HCrO_4_^−^, or Cr_2_O_7_^2−^ depending on the concentration and pH of the medium [9]. There are different methods for the partial or total recovery of Cr(VI) from water. The most common are electrocoagulation [10], microfiltration [11], membrane [12], photocatalytic reduction [13], catalytic reduction [14], and adsorption [15]. Most of these methods have complex and expensive procedures that should be considered when selecting the ideal treatment process. In contrast to other methods, adsorption is an economical and highly effective technique owing to its simplicity, straight forward design, and the ability to recover and recycle the contaminants [16]. The adsorption capacity of a system is largely dependent on the surface area available for adsorption, [17] and nanoparticles (NPs) are advantageous because have a very small size but a larger contact area than their bulk counterpart [18]. Various materials have been used as Cr(VI) adsorbents, such as steel industry waste material, rice husk ash, activated alumina, fuller’s earth, fly ash, saw dust, neem bark [19], and ferrites [20]. The solids that are generated after the adsorption process can be separated from the aqueous solution by centrifugation or filtration, but this implies additional cost and time of cleaning treatments [7]. Adsorption with magnetic NPs has been of great interest in recent years [21,22,23,24,25]. Magnetic materials offer the possibility of being easily extracted from contaminated areas by means of a magnet. The subsequent reuse after several adsorption/desorption cycles is also a good characteristic of NPs [20].

However, the use of magnetic NPs in the presence of an alternating magnetic field (AMF) generates heat [26]. The heating process can favor the adsorption of organic pollutants, as shown by a few authors [27,28]. It has been found that the application of a magnetic field increases the adsorption process, owing to the orientation of the adsorbent molecules against the sorbate, and the increase in the interaction between them in aqueous solutions [29]. Tireli et al. [27] used pillared clay and magnetic clay for the adsorption of methylene blue. They observed that the adsorbents had higher adsorption capacities in the presence of a magnetic field and that the highest capacity was with the magnetic clay. Zhang et al. compared the adsorption process between mycelial pellets that were adsorbing Congo red inside a thermostatic shaker heated by an AMF [28]. They found that the pellets heated inside the magnetic field had a higher adsorption rate compared to the pellets that were in the thermostatic shaker under the same temperature. Aigbe et al. reported an increase in the removal efficiency (RE) of Cr(VI) from an aqueous solution using a polypyrrole-based magnetic nanocomposite while increasing an unsteady electromagnetic field in the system [30]. These few studies focused on organic matter recovery, but there are no reports that focused on metal adsorption while applying a magnetic field.

In this study, magnetic NPs were synthesized via an electrochemical method, which is a scale-up process that allowed us to obtain 1 g of product per hour in a facile cleaning process [31]. The aim of this work was to evaluate the adsorption capacity of NPs as magnetic sorbents for Cr(VI) under three different conditions: at room temperature, with heating using a thermal bath, and heating under the presence of an external magnetic field.

## 2. Materials and Methods

### 2.1. Electrochemical Synthesis

The synthesis of Fe_3_O_4_ magnetic NPs was carried out in an electrochemical flow open cell, where eight electrodes were arranged in parallel, as described by Lozano et al. [31]. Iron electrodes were used as the anode and cathode with the same area of 48.6 cm^2^. The synthesis process was carried out in a 0.04 M NaCl solution used as electrolyte, and the current applied to the working electrode was 7.2 mA/cm^2^ using a PeakTech 1535 generator. The synthesis was performed at 25 °C for 7 h under constant recirculation of the electrolyte to obtain 7 g of NPs. With this method, it is possible to obtain a large number of NPs in a short period. After the synthesis, the NPs were washed three times with distilled water and dried at room temperature (RT) in vacuum conditions.

### 2.2. Material Characterization

The size and morphology of the synthesised NPs were analyzed by transmission electron microscopy (TEM) in a JEOL-1010 microscope (JEOL USA, Inc. Peabody, MA, USA) with an acceleration voltage of 100 kV. The NP size and size distribution were determined through the open source ImageJ software by using the TEM images and measuring the largest internal dimension of at least 300 NPs based on ISO 13322-1.

The magnetic properties of raw Fe_3_O_4_ NPs were determined using a vibrating-sample magnetometer MagLab VSM (Oxford Instruments plc, Oxford, UK) with a maximum field of 50 A/m. The samples were accurately weighed and fitted into the sample holder. A hysteresis loop of the powder samples was measured at 290 K up to ±3 kOe. With the resulting data, the values of saturation magnetization (MS), magnetic remanence (MR) in emu/gNP, and coercivity (HC) in Oe were obtained.

Zetasizer Nano ZS (Malvern Panalytical Ltd, Malvern, UK) was used to measure the zeta potential as a function of the pH at room temperature, using 0.01 M KNO_3_ as the electrolyte and HNO_3_ and KOH to adjust the pH.

X-ray photoelectron spectroscopy (XPS) was used to characterize the surface chemistry of the samples. XPS spectra were acquired at normal emission in an ultra-high vacuum chamber with a base pressure of 10-10 mbar equipped with a hemispherical electron energy analyzer SPECS Phoibos 150 spectrometer, (SPECS Surface Nano Analysis GmbH, Berlin, Germany) and a two-dimensional delay-line detector using Al and Mg-Kα (1486.6 and 1253.6 eV, respectively) X-ray sources operated at 200 W. Survey scans and specific core level spectra data were recorded with a pass energy of 20 eV, and energy steps of 0.5 and 0.05 eV, respectively [32]. Data processing was performed using the CasaXPS software (Casa Software Ltd., Cheshire, UK). The absolute binding energies of the photoelectron spectra were determined by referencing to C1s peak at 285.0 eV to correct any surface charging effect. The contributions of satellite peaks from non-monochromatic Al and Mg-Kα radiation in the region of Fe and Cr2p core levels were subtracted.

The concentration of chromium in the solution was evaluated before and after the adsorption tests by ultraviolet (UV) spectroscopy in a Perkin Elmer Lambda 35 UV-visible (UV-vis) spectrometer in the Cr(VI) maximum absorbance wavelength (350 nm) and by inductively coupled plasma optical emission spectroscopy (ICP-OES) using Perkin Elmer Optima 2100 DV, considering a wavelength of 220 nm. A calibration curve was obtained by preparing Cr(VI) stock solutions varying from 10 to 100 mg/L for the UV-vis analysis.

### 2.3. Adsorption Experiments

A 1000 mg/L stock solution of Cr(VI) was prepared by dissolving a specific amount of chromium oxide (VI) in 500 mL of distilled water. Working standards of desired Cr(VI) concentrations were prepared by diluting different volumes of the stock solution. The determination of RE of Cr(VI) using magnetic NPs was performed in a vessel containing 10 mL of contaminated solution at various ranges of metal concentrations and different amounts of sorbent (10, 20, and 30 mg of NPs). The solutions were under mechanical stirring (650 rpm) in all experiments. The effect of pH was analysed in the pH range between 2.5 and 6.5 at an initial Cr(VI) concentration of 25 mg/L. Afterwards, all chromium adsorption assays were carried out at an optimized pH of 3.5.

For the kinetic experiment, 20 mg of NPs was dispersed in a 25 mg/L Cr(VI) solution. The contact time was varied to 5, 10, 30, 60, and 120 min but the pH was kept constant at 3.5. After each adsorption time, the NPs were easily harvested from the solution by magnetic recollection and the absorbance of the supernatant was measured using the UV-vis spectrometer and ICP-OES. The results confirmed the reproducibility of the chromium concentration obtained with both techniques, with a deviation of approximately 0.5%. The appearance of iron dissolved in the solution due the acidic working pH in the adsorption experiments was not confirmed.

The RE of Cr(VI) from the contaminated solution and the equilibrium adsorption capacity (qe (mgCr(VI)/gNP)) of the NPs were calculated according to Equations (1) and (2), respectively:

(1) $$R_{E}\;\left(\%\right)=\frac{\left(C_{0}-C_{e}\right)}{C_{0}}\times 100$$

(2) $$q_{e}=\frac{\left(C_{0}-C_{e}\right)}{m}\times V$$

where $C_{e}$ is the equilibrium concentration of pollutant, in mg/L; $C_{0}$ is the initial pollutant concentration, in mg/L; m is the dry weight of adsorbent, in g; and V is the volume of pollutant solution, in L.

### 2.4. Magnetic Inductor Experiments

#### 2.4.1. Evaluation of the Maximum Heating Capacity of the Sorbent in Aqueous Media

The magnetic inductor used was Ambrell EASYHEAT LI 8310. The frequency and intensity were adjusted to 222 kHz and 350 A, respectively, and the increment of temperature was measured within 120 min. Such increment was varied depending on the concentration used. The sorbent concentration effect was evaluated in the range of 1–3 g/L. To proceed, the NPs were suspended in 10 mL of distilled water and the vessel was placed in the inductor under constant mechanical stirring at 650 rpm.

#### 2.4.2. Adsorption Test with Induction Heating

For the adsorption test, an aqueous solution with NPs was placed in the magnetic inductor until the set point of temperature was reached. Afterwards, an appropriate amount of a highly concentrated Cr(VI) solution was added to obtain the desired working concentration. The pH after the chromium addition was 3.5. After the adsorption tests, the sorbent was removed with a magnet and the equilibrium concentrations in the supernatants were analyzed.

### 2.5. Bath Experiments

To determine the effect of heating on the adsorption efficiency, bath experiments were carried out at the same temperature as that of the induction experiments. In this case, the Cr(VI) solution was heated in a thermal bath, and when the set temperature was reached, the NPs were added. The pH of the solution before the adsorption experiment was 3.5. The mechanical stirring and time were kept constant, i.e., 650 rpm and 2 h, respectively. All the experiments were conducted with 20 mg of sorbent and 10 mL of Cr(VI) aqueous solution to carry out the comparison between the heating methods employed.

### 2.6. Reusability Test

The determination of the reusability of NPs after the Cr(VI) sorption process was performed by analyzing the adsorption capacity of four successive sorption/desorption cycles. For this, 20 mg of NPs was mechanically mixed at 650 rpm with Cr(VI) solution (10 mL, C_0_ = 25 mg/L) at optimal conditions (2 h, pH 3.5) at room temperature. The chromium-loaded NPs were collected by magnetic harvesting after the sorption process, washed several times with distilled water, and then mixed with a solution of 0.2 M NaOH for 1 h at room temperature. Finally, the recuperated NPs were reused for each successive cycle and the Cr(VI) adsorption efficiency was obtained using Equation (1).

## 3. Results

**Figure 1:**
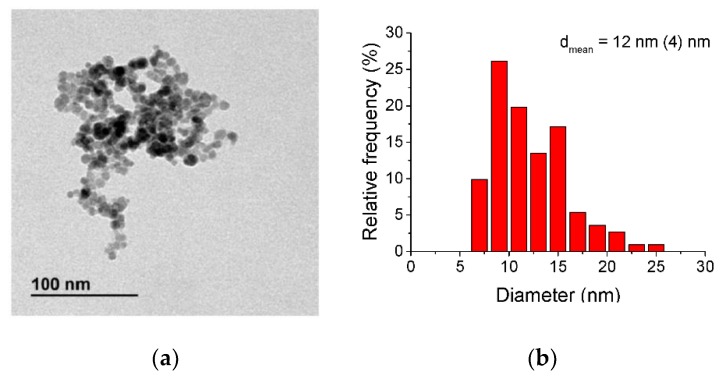
(**a**) Transmission electron microscopy (TEM) images of nanoparticles (NPs) electrochemically synthesised and (**b**) particle size distribution.

Figure 1 shows the TEM images, where the round-shaped NPs are shown with a mean diameter of 12 ± 4 nm (Figure 1b).

**Figure 2:**
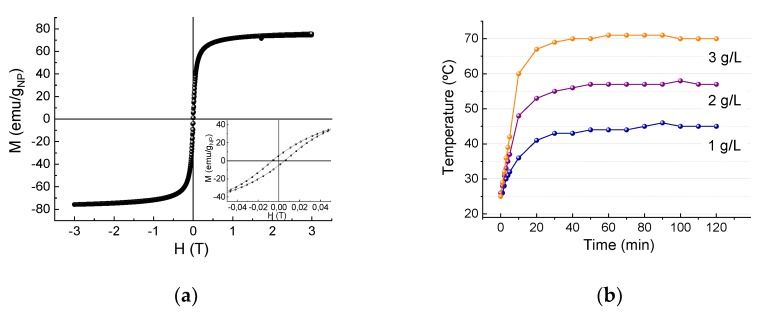
(**a**) Hysteresis loop of the sorbent at room temperature, inset is the zoom of the central area. (**b**) Temperature versus time plots at 1, 2, and 3 g/L nanoparticle concentration under the effect of a magnetic field (f = 222 kHz, I = 350 A).

The use of magnetic NPs for the adsorption of pollutants was based on two principal reasons. First is the capacity of NPs to be extracted from a contaminated area by magnetic harvesting and second is the heating capacity of NPs when they are exposed to an AMF [33]. The temperature gradient of an NP under an AMF diminishes exponentially with the distance to the surface, becoming more marked in the first nanometers [34]. This behavior in the vicinity of the surface is completely different from the global increment observed in an NP colloidal solution, where the global temperature is the average temperature of all the solution and is always lower. The magnetic heating capacity of superparamagnetic NPs was described by the linear response theory predicted by the literature [35], where it is assumed that the heat generated is mainly determined by the effective relaxation time of the NPs and the magnetic field and frequency applied. The magnetic anisotropy and saturation magnetization values affect the heating dissipation process. In this context, the magnetic hysteresis loop of the sorbent (Figure 2a) confirms the superparamagnetic characteristic of the Fe_3_O_4_ electrochemical sample, with almost null coercivity and remanence values of 65 Oe and 5 emu/g, respectively. The saturation magnetization of that sample is 75 emu/g lower than the Ms bulk value, probably due to NP superficial effects, and comparable or similar to that of other magnetite NPs reported with same size [36]. To verify the heating abilities of the NPs, different colloidal solutions, varied within a concentration range of 1–3 g NPs/L, were exposed to the induction heater. The maximum temperature set values for the different concentrations in the thermal bath experiments were fixed. These values were 45, 60, and 70 °C for concentrations of 1, 2, and 3 g/L, respectively (see Figure 2b).

### 3.1. Experimental Concerns on the Adsorption Process

The remediation of certain aqueous pollutants when solid sorbents are used depends on many operational factors, such as pH, quantity of sorbate, contact time, and solution temperature. All these enumerated parameters were analyzed to determine the most operative experimental conditions.

#### 3.1.1. Effect of pH

**Figure 3:**
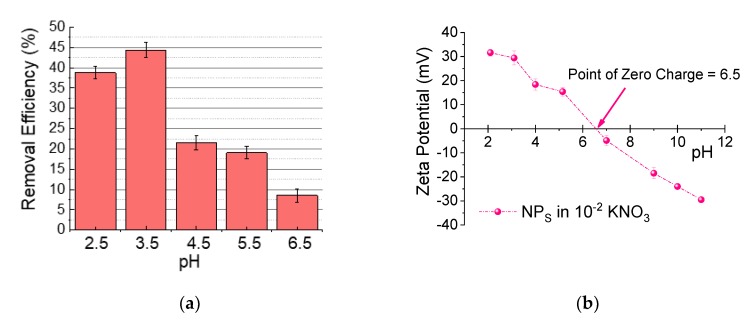
(**a**) Effect of the pH batch solution in the adsorption capacity in equilibrium when work with 1 g/L sorbent concentration and 25 mg/L of adsorbate. (**b**) Zeta potential of nanosorbent as a function of pH. Dots are connected to guide the eyes.

The surface charge of a sorbent in aqueous media plays an important role in the adsorption process. pH is a key factor in adsorption because it alters the functional groups in sorbents and the ionic state of pollutants. In the case of chromium, removal is mostly evaluated in acidic media, when the surface of the pollutant is negatively charged and the predominant species is (HCrO_4_)^−^ [37]. Therefore, to correlate these facts, the zeta potential and adsorption efficiency as a function of pH of bare NPs were explored (Figure 3). The adsorption capacities as a function of pH for the nanosorbent are shown in Figure 3a. As can be seen, higher extraction efficiencies were obtained in the pH range of 2.5–3.5, followed by a decrease with a rise in pH. Hence, it is evident that the adsorption process is highly dependent on the pH. A small modification in the pH affects the surface charge of the adsorbent and the different speciations and/or proportions of adsorbate. At pH < 6.5, the Cr in the solution is the anion bichromate (HCrO_4_^−^) and at pH > 6.5 it is the anion chromate (CrO_4_^2−^). Therefore, considering the electrostatic interaction between the adsorbate and adsorbent, the positive charge of the adsorbent at the working pH facilitates the adsorption process (see Figure 3b). Contrary to this, the reproducibility of the experiment and the reusability of the NPs at pH 2.5 decreased. In this case, in the UV-vis spectra (not shown), a flat band appeared at 700 nm and rose as the wavelength decreased. The partial dissolution of the iron oxide in the acidic environment contributed to a false increase in the chromium peak in 350 nm. However, at pH 3.5, the UV measurement of the supernatant does not show the iron contribution; thus, eventually, pH 3.5 was selected for further exploration to assure the maximum adsorption capacity, reproducibility of the experiment, and reusability of the sorbents. At that working pH the zeta potential was about +20 mV, high enough to assure the colloidal stability during the adsorption test.

#### 3.1.2. Effect of Sorbate Dose

**Figure 4:**
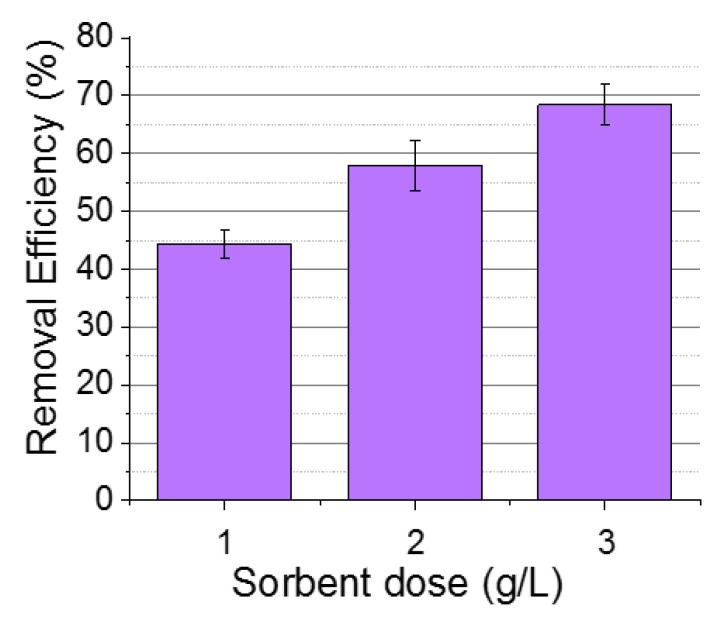
Effect of the sorbent dose in the removal efficiency of NPs (C_0_: 25 mg/L; pH 3.5).

The effect of sorbent dose, which is an important parameter, was also investigated to determine the relationship between chromium adsorption and nanosorbent mass. Figure 4 depicts the RE when working with sorbent doses in the range of 1–3 g/L. It is evident from Figure 4 that the adsorption removal percentage of Cr(VI) increases with the sorbent dose increment; however, it is not proportional to the mass used. That is, the relationship between the adsorption efficiency and the presence of more active surface sites (which are available to interact with the pollutant) with the increment in mass is not linear. The magnetic aggregation of the NPs at higher concentrations can contribute to diminishing the availability of these active sites, and therefore, a decrease in efficiency was observed. To avoid the aggregation effect and achieve maximal adsorptive efficiencies, the absorbent dose of 2 g/L was selected to evaluate the influence of the heating method on the Cr(VI) elimination.

#### 3.1.3. Kinetic Adsorption Process

The contact time was investigated by performing adsorption tests at optimum conditions and varying it from 5 min to 2 h. The equilibrium adsorption capacity was calculated for each sample by fitting the experimental data to pseudo-first order and pseudo-second order models, i.e., Equations (3) and (4), respectively:

(3) $$q_{t}=q_{e}\left(1-e^{-k_{1}t}\right)$$

(4) $$q_{t}=\frac{k_{2}q_{e}^{2}t}{1+k_{2}q_{e}t}$$

**Figure 5:**
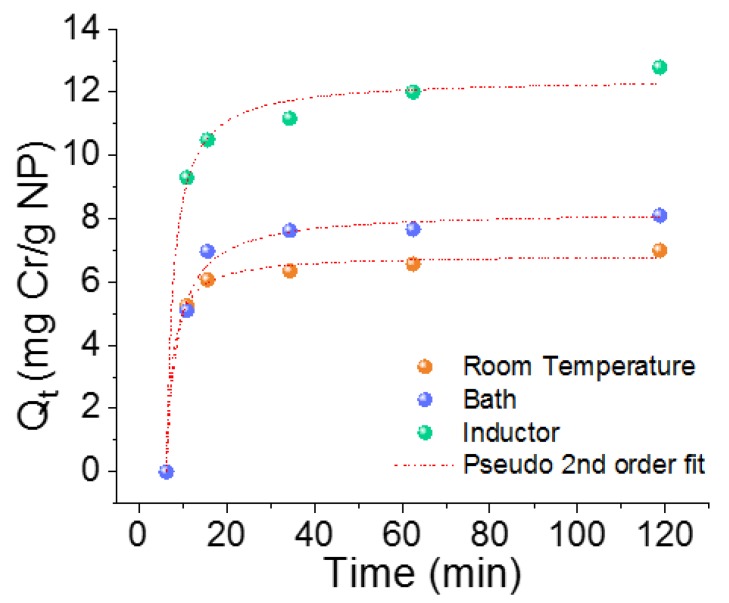
Time dependence of adsorption capacity of Cr(VI) ions onto NPs at room temperature and at 60 °C, in thermal bath and in inductor conditions with a sorbent dose of 2 g/L.

The equilibrium adsorption capacity at room temperature was reached at 2 h of contact time. The same contact time was obtained for the batch experiments in the inductor and bath. Table 1 summarises the fitted kinetic parameters obtained for the different experiments. The experimental data best fitted the pseudo-second order model with a good determination coefficient (R^2^) in all cases. Because of this, it can be assumed that the rate-limiting step is the surface adsorption, and the Cr(VI) removal is due to the physicochemical interaction with the sorbent. Figure 5 shows the fitting of NPs to the pseudo second-order kinetic model at room temperature, bath, and inductor experiments.

So far, the pH of the solution, contact time, and sorbent dose have been analyzed and optimized to achieve the maximal adsorption yield. Next, we focused on evaluating the removal of Cr(VI) using different heating modes, namely thermal bath and magnetic heater.

### 3.2. Evaluation of Induction Heating Effect on the Removal of Cr(VI)

Although some studies have shown that high temperature adversely affects the adsorption of Cr(VI) [38], in most of the studies, an increase in adsorption was observed [21,39,40]. None of these studies focused on the different ways or modes of heating that can be applied to a sorbent to increase the system temperature. Generally, the existing usual mode of heating is by immersion in a thermal bath and heating by magnetic induction. The latter relies on the capability of the magnetic dipole of NPs to couple to an AMF and on the subsequent dissipation of the absorbed energy by its release as heat [41]. Induction heating has proved to be very useful for magnetic fluid hyperthermia, which promotes cell death [42]. More recently, it is starting to be used in in situ polymerisation of NP surfaces [43] or even for synthesising organic molecules [44].

To evaluate the effect of the mode of heating used on the RE, kinetic studies with bare NPs were carried out in a thermal bath and in a magnetic inductor. In both cases, the pollutant was added when the NP aqueous solution had reached the desired temperature. That temperature was estimated when magnetic induction heating was applied to aqueous sorbent solutions at different concentrations (see Figure 2b). The temperature was fixed at 60 °C in the case of working in a 2 g/L NP concentration. Adsorption test at room temperature was added as a control.

**Figure 6:**
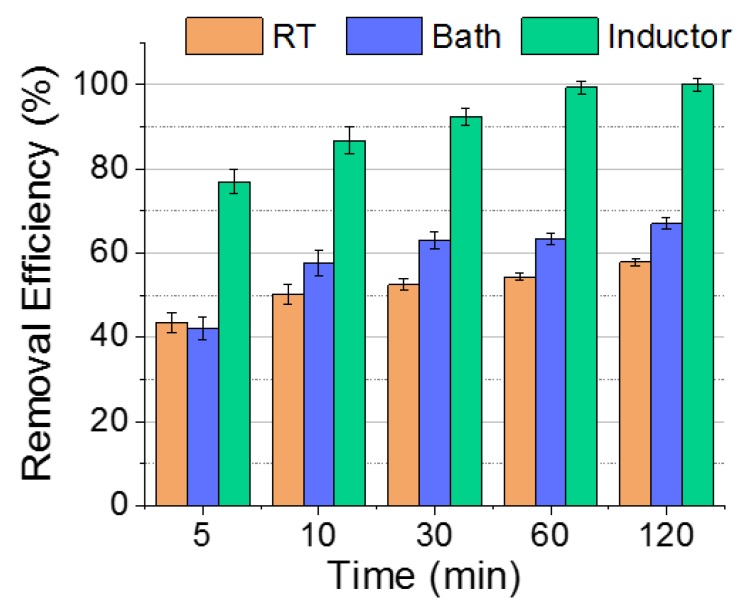
Removal efficiency of bare nanoparticles when the sorption experiment has been performed at room temperature, and at 60 °C in a thermal bath and under inductor heater with a 25 mg/L of pollutant concentration.

Figure 6 shows the adsorption efficiencies of bare NPs at room temperature (brown), heating bath at 60 °C (blue), and heating by magnetic field at 60 °C (green). Initially, because chromium adsorption on a sorbent is an endothermic process [45], the RE was higher at 60 °C for the bath and inductor cases than at room temperature. Aside from this, the NPs that were under the presence of the magnetic field showed greater adsorption capacity than the ones that were in the thermal bath. Even though both heating systems were at the same global temperature (T_global_), the temperature of the NP surface and the surroundings when they are under the presence of an AMF is much greater and decreases exponentially with the distance. Some authors had assessed the local temperature (T_local_) increment in the surroundings of the NP surface in comparison with the net global temperature. For example, Riedinger et al. [46] had developed a thermosensitive probe to assess the absolute temperature at distances of up to 0.5 nm from the surface. They claimed that the local temperature could decrease up to 70 °C at 5 nm distance from the NP surface. Dias et al. [47] had also developed a thermal probe based on the denaturalisation of the DNA grafted in the NP surface with the temperature. They demonstrated that when the global temperature is 33 °C, the temperature at 5 nm away from the NP surface is approximately 42 °C. For that reason, if T_local_ > T_global_, it may lead to an improvement in the adsorption capacity.

Therefore, in general, the adsorption efficiency is almost doubled under a magnetic inductor in comparison with room temperature conditions. However, the most interesting finding was the impressive reduction in the time required to adsorb certain Cr(VI) concentrations by applying magnetic field heating. Only 5 min was required to achieve a greater efficiency compared to the 2 h of contact time at 60 °C in the case of thermal bath exposure.

**Figure 7:**
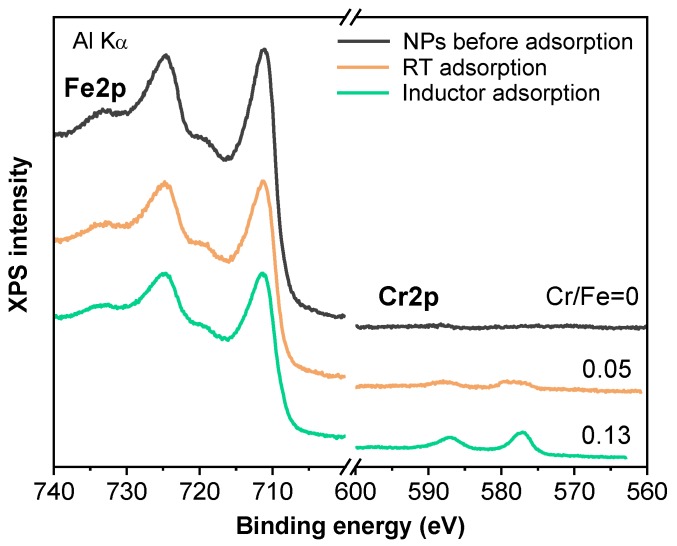
Fe2p and Cr2p XPS spectra taken with Al-Kα radiation corresponding to NPs before adsorption, and room temperature and inductor processed samples. Relative percentage of Cr/Fe atomic concentration determined by XPS analysis is shown.

To evaluate the presence of chromium atoms in the NP surface and the oxidation state, XPS measurements were performed. Figure 7 displays the intensity evolution of Fe2p and Cr2p spectra corresponding to each sample. The increase in Cr signal when an external magnetic inductor was used in the adsorption experiments is readily seen. The overall Cr/Fe surface composition ratio was determined from the evaluation of their individual element regions. The integral peak areas for each element, after background subtraction and normalization using sensitivity factors provided by the electron energy analyzer manufacturer, were used to calculate the relative atomic concentration of the samples. There was a threefold increase in the Cr adsorption rate when the inductor was used, as shown in Figure 7.

**Figure 8:**
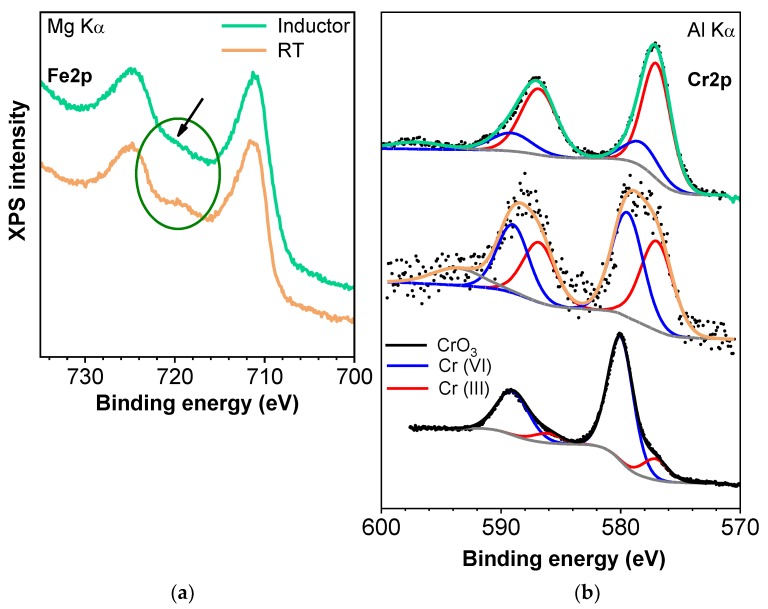
(**a**) Comparison of the normalized Fe2p X-ray photoelectron spectroscopy (XPS) spectra taken with Mg-Kα radiation corresponding to the absence and presence of magnetic inductor in the Cr adsorption experiments. (**b**) Normalized Cr2p XPS spectra taken with Al-Kα radiation from samples processed with/without inductor together with CrO_3_ reference. Deconvolution of Cr2p spectra with fit composed of chemically shifted components associated to VI and III oxidation states. Data points in every spectrum are represented as black symbols, and Shirley background and component peaks using solid lines.

Some authors have reported the reduction of Cr(VI) to Cr(III) after adsorption [48]. They detected this process using magnetite NPs functionalised with humic acid by means of extended X-ray absorption fine structure measurements. Moreover, using only magnetite NPs, they detected that the adsorption mechanism includes electron transfer between Cr(VI) and Fe(II) for the possible formation of Cr_x_Fe_(1−x)_(OH)_3_ [49]. A detailed analysis of the Fe2p spectra taken with Al-Kα radiation reveals no significant difference in the line shape for the three types of samples; in fact, superposition of all the spectra confirms a perfect overlapping of not only the main double peaks but also the satellite features. In addition, the spectra are consistent with the existence of a rich Fe(III) oxidation state on the surface of the Fe_3_O_4_ NPs. At first, one might consider the possibility of no chemical changes on the iron oxide NP surface in the Cr adsorption experiments. However, the analysis of Cr2p emissions indicates a clear modification in the line shape (refer to the next discussion). This controversial result can be understood by considering, on one hand, the small amount of Cr adsorbed on the NPs surface and, on the other, the depth sensitivity of XPS, which is high and probes also the inner layers of the NPs when spectra are measured with Al-Kα radiation. To clarify this situation, further XPS experiments were also performed with a Mg-Kα anode, which provides a higher surface sensitivity owing to the lower photon energy. Therefore, any variation in the oxidation state on the outermost layers of the iron oxide NPs will be enlarged. Figure 8a displays the Fe2p signals corresponding to room temperature and inductor-processed samples. Even though at first sight both spectra look very similar, a close inspection of the binding energy region between the doublet indicates a very subtle emission that shifts and increases the satellite characteristic of Fe(III) (marked by an arrow in the figure) together with a decrease in Fe(II) satellite present on the left shoulder of the Fe2p3/2 peak. This result might be associated with the increase in Fe(III) state at the expense of Fe(II) due to the interaction of Cr(VI) and Fe_3_O_4_. This effect is very weak because it is limited to the interface region of chromium and iron oxide. The analysis of the corresponding Cr2p also supports the expected partial reduction of chromium from Cr(VI) to Cr(III) at the chromium/iron oxide interface by taking into account electrochemical potential considerations.

Figure 8b shows the Cr2p core level peaks of RT/inductor-processed samples together with the spectrum of CrO_3_ used in the adsorption experiments, which serves as a reference for comparison and for determining the different Cr oxidation states present in the samples. The Cr2p spectra have been normalised to the maximum peak intensity in each case for better visual inspection and to compare the oxidation state ratios depending on the preparation process of the samples. From the direct comparison of the spectra, one can readily observe line shape differences, which were enhanced, first by the broadening of the low binding energy side of the Cr2p peaks, and second by their continuous shift to lower values and the corresponding peak narrowing. This effect is a consequence of the Cr chemical reduction evolution.

In addition, for a detailed analysis of the reduction process and to evaluate the presence of oxidation states of the samples depending on the preparation process, Cr2p spectra fitting was performed by the deconvolution of several mixed percentage of Gaussian–Lorentzian symmetric functions. The energy of the peaks and their relative heights were determined by the least-squares method to account for the emission ascribed to the different chemical environments according to the binding energy values reported [50,51,52]. In particular, Figure 8b displays the Cr2p core level from the CrO_3_ reference sample (lower spectrum) with its fit composed of chemically shifted components associated with Cr(VI) and Cr(III) oxidation states. The peak assignment was based on the binding energy values in comparison with the reported ones for those oxidation states. The fitting curve provides a majority signal mainly formed by Cr(VI), as expected. There was a minor Cr(III) contribution that came from the outermost surface layers of the CrO_3_ sample. This effect was confirmed by take-off emission XPS experiments, in which the shoulder (component) associated with Cr(III) suffered a weak but continuous increase in the grazing emission detection geometry. In the case of the RT-processed sample, Cr(VI) and Cr(III) states were balanced despite the small Cr content. However, the upper spectrum was basically fit with an intense component ascribed to Cr(III) supporting the strong effect produced by the induction method, which yields satisfactory results in terms of chemical reduction.

### 3.3. Desorption and Reusability

**Figure 9:**
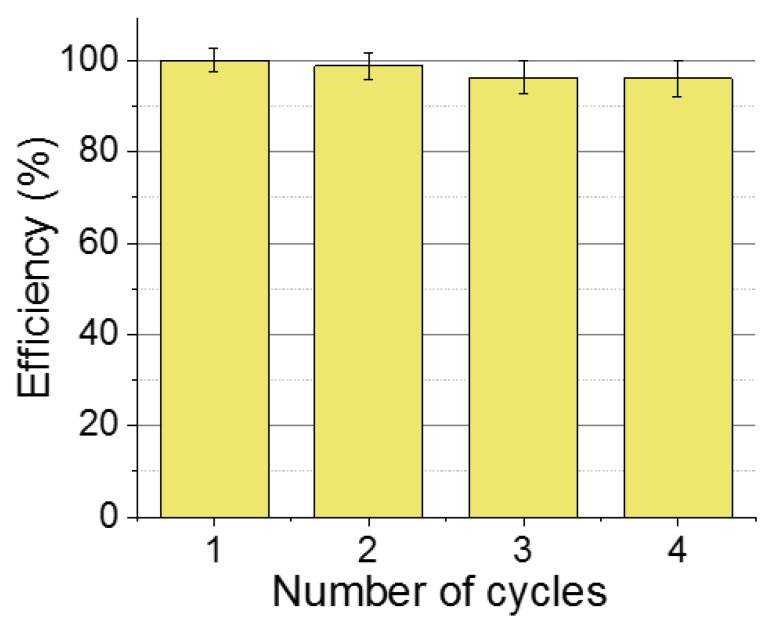
NPs reusability tests for chromium (VI) removal.

It was demonstrated that Cr(VI) has a special affinity to be attached to the NP surface, but for a cost-effective adsorbent, it is essential to study the reusability of the system. The removal of Cr(VI) ions was carried out following the report [21]. Figure 9 shows the recycle graph. After four cycles, there was a slight activity decrease in the efficiency values of approximately 4%, that is, under the error bar estimated. The morphology and the magnetic characteristics of the NPs after regeneration were not altered. Therefore, this material can be used at maximum performance for at least four cycles.

## 4. Conclusions

Magnetite NPs were successfully produced by a simple electrochemical method, which generated a considerable number of NPs in less time with easier cleaning processes than other methods. These NPs were used as adsorbents of Cr(VI) from aqueous solutions. The maximal adsorption capacities were obtained when working at pH 3.5, with a contact time of 2 h and with 2 g/L of sorbent. The NPs were then heated under the presence of an external magnetic field and the results indicated that the removal of Cr(VI) is highly dependent on the heating mode. The magnetic inductor reduced the time to only 5 min to obtain the same amount of chromium removal for 2 h when working at room temperature.

The XPS results verified the chemical reduction of Cr(VI). These results confirmed not only the convenience of using an external magnetic inductor for Cr adsorption enhancement but also its benefits for the chemical reduction from Cr(VI) to Cr(III). Both advantages support this procedure as an alternative method for the efficient removal of Cr(VI) in contaminated CrO_3_ solutions, which is important progress in terms of practical environmental applications. Interestingly, the ability of the adsorption of Cr(VI) in the presence of magnetic induction heating opens up the way to apply this technique to additional pollutants as well as to pollutant mixture.

## Figures and Tables

**Table 1:** Kinetic constants of NPs performed under three different experimental conditions (room temperature (RT), bath immersion, and inductor heating) at 2 g/L sorbent concentration.

Constant	Units	NPs		
		RT	Bath	Inductor
q_e_	mg/g	6.6(1)	7.8(1)	11.9(4)
k_1_	min^−1^	0.30(4)	0.21(1)	0.28(5)
R^2^		0.991	0.997	0.982
q_e_	mg/g	6.9(1)	8.2(2)	12.4(3)
k_2_	g/mg	0.10(2)	0.046(9)	0.045(9)
R^2^		0.996	0.991	0.993
